# Supplementary material for: COVID-19 vaccine coverage among adults in Sarlahi District of Nepal in 2022
Source: PLOS Glob Public Health. 2025 May 15;5(5):e0003916. doi: 10.1371/journal.pgph.0003916 (PMC12080811; doi:10.1371/journal.pgph.0003916)
Supplement: S1 Table — (DOCX) [file pgph.0003916.s001.docx]

**S1 Table: Vaccination accessibility questions among community members**

| **Vaccination accessibility questions** | **Overall (n=362)** |
| --- | --- |
| **Is the health post for vaccination far?** |  |
| Yes | 115 (31.8%) |
| No | 245 (67.7%) |
| Do not know | 2 (0.6%) |
| **Vaccination wait is too long?** |  |
| Yes | 208 (57.5%) |
| No | 151 (41.7%) |
| Do not know | 3 (0.8%) |
| **Know if there is vaccine in your health post currently?** |  |
| Yes | 22 (6.1%) |
| No | 85 (23.5%) |
| Do not know | 255 (70.4%) |
| **Will you lose income by going to vaccination clinic?** |  |
| Yes | 120 (33.1%) |
| No | 242 (66.9%) |
| Do not know | 0 (0%) |
| **Will you have more personal expense on your way to get the vaccine than usual?** |  |
| Yes | 77 (21.3%) |
| No | 285 (78.7%) |
| Do not know | 0 (0%) |
