# Supplementary material for: COVID-19 vaccine coverage among adults in Sarlahi District of Nepal in 2022
Source: PLOS Glob Public Health. 2025 May 15;5(5):e0003916. doi: 10.1371/journal.pgph.0003916 (PMC12080811; doi:10.1371/journal.pgph.0003916)
Supplement: S2 Table — (DOCX) [file pgph.0003916.s002.docx]

**S2 Table: COVID-19 vaccine brand combinations**

| **Brand combinations**  **(Dose 1 - Dose 2 - Dose 3)*** | **One-dose only (n=51)** | **Primary-series (n=188)** | **Boosted (n=82)** | **Unvaccinated (n=41)** | **Overall (n=362)** |
| --- | --- | --- | --- | --- | --- |
| AstraZeneca-AstraZeneca-AstraZeneca | 0 (0%) | 0 (0%) | 23 (28.0%) | 0 (0%) | 23 (6.4%) |
| Sinopharm-Sinopharm-Sinopharm | 0 (0%) | 0 (0%) | 10 (12.2%) | 0 (0%) | 10 (2.8%) |
| Pfizer-Pfizer-Pfizer | 0 (0%) | 0 (0%) | 1 (1.2%) | 0 (0%) | 1 (0.3%) |
| Moderna-Moderna-Moderna | 0 (0%) | 0 (0%) | 1 (1.2%) | 0 (0%) | 1 (0.3%) |
| AstraZeneca-AstraZeneca-Moderna | 0 (0%) | 0 (0%) | 4 (4.9%) | 0 (0%) | 4 (1.1%) |
| Sinopharm-Sinopharm-AstraZeneca | 0 (0%) | 0 (0%) | 17 (20.7%) | 0 (0%) | 17 (4.7%) |
| Sinopharm-Sinopharm-Moderna | 0 (0%) | 0 (0%) | 9 (11.0%) | 0 (0%) | 9 (2.5%) |
| Sinopharm-AstraZeneca-AstraZeneca | 0 (0%) | 0 (0%) | 1 (1.2%) | 0 (0%) | 1 (0.3%) |
| CoronaVac-CoronaVac-Pfizer | 0 (0%) | 0 (0%) | 1 (1.2%) | 0 (0%) | 1 (0.3%) |
| Sputnik v-Sputnik v-Pfizer | 0 (0%) | 0 (0%) | 1 (1.2%) | 0 (0%) | 1 (0.3%) |
| J&J-J&J-NA | 0 (0%) | 0 (0%) | 2 (2.4%) | 0 (0%) | 2 (0.6%) |
| J&J-AstraZeneca-NA | 0 (0%) | 0 (0%) | 10 (12.2%) | 0 (0%) | 10 (2.8%) |
| J&J-Sinopharm-NA | 0 (0%) | 0 (0%) | 1 (1.2%) | 0 (0%) | 1 (0.3%) |
| J&J-Unknown-Unknown | 0 (0%) | 0 (0%) | 1 (1.2%) | 0 (0%) | 1 (0.3%) |
| J&J-NA-NA | 0 (0%) | 34 (18.1%) | 0 (0%) | 0 (0%) | 34 (9.4%) |
| AstraZeneca-AstraZeneca-NA | 0 (0%) | 54 (28.7%) | 0 (0%) | 0 (0%) | 54 (14.9%) |
| Sinopharm-Sinopharm-NA | 0 (0%) | 84 (44.7%) | 0 (0%) | 0 (0%) | 84 (23.2%) |
| CoronaVac-CoronaVac-NA | 0 (0%) | 1 (0.5%) | 0 (0%) | 0 (0%) | 1 (0.3%) |
| Moderna-Moderna-NA | 0 (0%) | 1 (0.5%) | 0 (0%) | 0 (0%) | 1 (0.3%) |
| AstraZeneca-Sinopharm-NA | 0 (0%) | 2 (1.1%) | 0 (0%) | 0 (0%) | 2 (0.6%) |
| Sinopharm-AstraZeneca-NA | 0 (0%) | 4 (2.1%) | 0 (0%) | 0 (0%) | 4 (1.1%) |
| Unknown-AstraZeneca-NA | 0 (0%) | 1 (0.5%) | 0 (0%) | 0 (0%) | 1 (0.3%) |
| Unknown-Moderna-NA | 0 (0%) | 1 (0.5%) | 0 (0%) | 0 (0%) | 1 (0.3%) |
| Unknown-Unknown-NA | 0 (0%) | 6 (3.2%) | 0 (0%) | 0 (0%) | 6 (1.7%) |
| AstraZeneca-NA-NA | 29 (56.9%) | 0 (0%) | 0 (0%) | 0 (0%) | 29 (8.0%) |
| Sinopharm-NA-NA | 15 (29.4%) | 0 (0%) | 0 (0%) | 0 (0%) | 15 (4.1%) |
| Moderna-NA-NA | 5 (9.8%) | 0 (0%) | 0 (0%) | 0 (0%) | 5 (1.4%) |
| Unknown-NA-NA | 2 (3.9%) | 0 (0%) | 0 (0%) | 0 (0%) | 2 (0.6%) |
| NA-NA-NA | 0 (0%) | 0 (0%) | 0 (0%) | 41 (100%) | 41 (11.3%) |
| *NA = Not applicable (dose not received) | | | | | |
