# Supplementary material for: COVID-19 vaccine coverage among adults in Sarlahi District of Nepal in 2022
Source: PLOS Glob Public Health. 2025 May 15;5(5):e0003916. doi: 10.1371/journal.pgph.0003916 (PMC12080811; doi:10.1371/journal.pgph.0003916)
Supplement: Attachment — Submitted filename: Article suggestions.docx [file pgph.0003916.s004.docx]

Respected Editor,

I am grateful for the opportunity to review the manuscript. Please find my suggestions below.

**Brief background**

The study titled "COVID-19 Vaccine Coverage in Sarlahi District of Nepal" assessed COVID-19 vaccine uptake and its associations with participant characteristics in a rural, low-resource setting.

**Comments**

**Title**

1. Population of interest (rural adults/children), timeline component and type of study should preferably be mentioned in the title.

**Abstract**

1. Methods – Repeating of variables is there (for example: Vaccine card availability is repeated twice); Instead something like “Data was summarized using mean (SD) and frequency with proportions” could be mentioned. And the word “risk factors” is not appropriate here. Better to frame it as “factors associated with”

**Introduction**

1. Justification for the methodology adopted in the study needs to improve - why assessing COVID – 19 vaccine coverage in the first place? Why restricted only to rural area? Why coverage in children not assessed?
2. References needed for the following paragraph – “As vaccine access increased, demand and uptake came to depend not just on access but also on perceived risks and vaccine hesitancy. It is important to maintain coverage to prevent outbreaks given emerging SARS-CoV-2 variants and the continued risks for certain vulnerable populations. such as the elderly and pregnant women.”

**Methods**

1. Time period – Can mention the timeframe only. The statement “after the spillover of the Delta variant infections from neighboring India and escalating mortality (14,15)” can be shifted to discussion.
2. Sampling –

- How did the authors sample these four nagarpalikas/gaunpalikas from the entire Sarlahi district? Is it representative of the entire district?
- How the households were selected from each ward?
- Better to show the multistage cluster sampling which has been adopted using a flowchart with sampling technique adopted at every stage from the district to the individual level.

3. Sample size –

- What is the estimated prevalence for vaccine coverage (not vaccine hesitancy) taken for the study? Prevalence should be 50% (not 0.5).
- What is the absolute/relative precision used?
- Kindly cross check the calculation and rewrite it appropriately.

4. Recruitment and consenting –

- Could the timing of data collection have influenced which gender is more likely to be recruited? (females are more here)
- Were the questions self administered or interviewer administered?
- Whether efforts were taken to include those that were “Not met (n=32)”
- The writing in this section has scope of improvement with respect to grammar and by avoiding repetitions of sentences.

5. Measurement and definitions –

- Why was it decided to categorize vaccination status as partially if other brands were administered? Kindly provide justification for the classification used. If the participants didn’t know the brand, how were they classified?
- Some of the parameters used to calculate vaccination accessibility don’t belong to this category (For example: “being aware if the nearest health facility had vaccine stock” comes under knowledge more than accessibility). Was this scale derived from a standard tool? If yes, provide details for the same.

6. Data management and analysis –

- Kindly mention how descriptive analysis was done – mean (SD) or frequency with proportions
- For inferential statistics – 95% CI and p value (how much is significant)
- Regression – Better to report prevalence odds rather than odds ratio as this is a cross sectional study

**Results**

1. Table 1: The variables “Water source/latrine type”, etc are already accounted for in wealth quartiles. Mentioning these variables separately might not be appropriate while seeing for difference between completed vs incomplete primary series. If the author wants to retain these variables, they can create a separate table for basic socio-demographic characteristics and mention it there without comparing completed vs incomplete primary series. They can then include a few selected variables for the comparison aspect.
2. Justification for including table 2 (Is there a need to highlight a separate table for vaccine card availability?)
3. Table 4: – Better to report prevalence odds; How was social media usage measured? Has this variable been mentioned in earlier tables?

**Discussion**

1. There is scope of improvement in the structuring and flow of content in discussion.
2. Details related to vaccine accessibility could be discussed with respect to other studies
3. Content related to country of Bhutan can be cut short and relevant points to your study could be retained
4. Other biases with respect to sampling and measurement could be elaborated and its implication discussed.
