# Supplementary material for: COVID-19 vaccine coverage among adults in Sarlahi District of Nepal in 2022
Source: PLOS Glob Public Health. 2025 May 15;5(5):e0003916. doi: 10.1371/journal.pgph.0003916 (PMC12080811; doi:10.1371/journal.pgph.0003916)
Supplement: Attachment — Submitted filename: Response to Reviewers_March7 .docx [file pgph.0003916.s006.docx]

| **Reviewer comments** | **Changes** |
| --- | --- |
| **Reviewer 1** |  |
| **The study which is descriptive is simple and easy to understand. The study evaluates COVID-19 vaccination coverage and determinants that could be relevant in similar vaccine preventable epidemics. The authors also outlined the limitations of the study. I think it may have added value to the study if the occurrence of COVID-19 infection among the study participants was investigated to determine vaccination effectiveness.** | Out study design will not have allowed to estimate effectiveness. |
| **Reviewer 2** |  |
| **Title**   1. Population of interest (rural adults/children), timeline component and type of study should preferably be mentioned in the title. | Line 1:  The title has been changed to: COVID-19 Vaccine Coverage Among Adults in Sarlahi District of Nepal in 2022 |
| **Abstract**   2. Methods – Repeating of variables is there (for example: Vaccine card availability is repeated twice); Instead something like “Data was summarized using mean (SD) and frequency with proportions” could be mentioned. | The repetition was intentional to say what was collected during the survey and what was also used in descriptive analyses. |
| 3. And the word “risk factors” is not appropriate here. Better to frame it as “factors associated with” | Line 35:  “…analyze factors associated with completing the primary vaccination series.” |
| **Introduction**   4. Justification for the methodology adopted in the study needs to improve - why assessing COVID – 19 vaccine coverage in the first place? Why restricted only to rural area? Why coverage in children not assessed? | Lines 95-96:  We mention the reason for assessing coverage in:  lines 95-96: “Rigorous studies have not estimated vaccine coverage in rural Nepal, especially the Terai region.”  During this time of the pandemic, vaccinations had only just been recommended for children. The program had not fully rolled out, so it would have been premature to measure coverage among children at that time. |
| 5. References needed for the following paragraph – “As vaccine access increased, demand and uptake came to depend not just on access but also on perceived risks and vaccine hesitancy. It is important to maintain coverage to prevent outbreaks given emerging SARS-CoV-2 variants and the continued risks for certain vulnerable populations. such as the elderly and pregnant women.” | Line 80-81:  “Vaccine demand and uptake also came to depend on perceived risks and vaccine hesitancy (9,10).” |
| **Methods**  **Time period**   6. Can mention the timeframe only. The statement “after the spillover of the Delta variant infections from neighboring India and escalating mortality (14,15)” can be shifted to discussion. | Moved to line 303-304 |
| **Sampling**   7. How did the authors sample these four nagarpalikas/gaunpalikas from the entire Sarlahi district? Is it representative of the entire district? | Please see limitations section in discussion:  Line 304-310: “The study was conducted in four municipalities, which roughly approximate the larger Terai region, but is not necessarily representative of Nepal overall. This study utilized a pre-existing census from the parent randomized trial as the sampling frame that comprised households that had a woman 15-30 years of age. Hence, our study households may not reflect all households in the district, although most households, which include multigenerational families, have women of childbearing age” |
| 8. How the households were selected from each ward? | Line 108-112:  “This study had previously constructed a population-based list of households in six Nagarpalikas and Gaunpalikas of rural Sarlahi District, Nepal, in which married females, 15-30 years of age resided. We randomly selected households from this census, proportional to the ward-level population size, from 17 wards in 4 Nagarpalikas and Gaunpalikas— Haripur, Ishworpur, Kabilasi and Chandranagar (Figure 1).” |
| 9. Better to show the multistage cluster sampling which has been adopted using a flowchart with sampling technique adopted at every stage from the district to the individual level. | We did not use a multistage cluster sampling approach. Instead, we purposively selected 17 wards from 4 Nagarpalikas and Gaunpalikas, which cover a large, contiguous area in our parent study site. Then, we randomly selected households from each ward in proportion to each ward’s population size. Therefore we don’t feel that a flowchart would help explain this sampling process, given it is not multistage cluster sampling. |
| **Sample size**   10. What is the estimated prevalence for vaccine coverage (not vaccine hesitancy) taken for the study? Prevalence should be 50% (not 0.5). | Line 112-114:  Given that vaccine hesitancy and coverage were estimated to be about 50% for purposes of sample size, we have modified the sentence clarifying this in line 112-114 “We selected a sample size of 362 participants to allow for a two-sided 95% confidence interval with a width equal to 0.12 around an assumed sample prevalence of vaccine coverage of 50% (17). |
| 11. What is the absolute/relative precision used? | Line 114:  “…around an assumed sample prevalence of vaccine hesitancy of 50% (17).” |
| 12. Kindly cross check the calculation and rewrite it appropriately. | We made minor edits to this in the text. The calculation was double checked and is correct. |
| **Recruitment and consenting**   13. Could the timing of data collection have influenced which gender is more likely to be recruited? (females are more here) | Yes, as during the day time men are at work. Please see line 311. |
| 14. Were the questions self administered or interviewer administered?     a. 2 interviewers in local Maithili an Nepali language | Interviewer administered.  Changed the following in Line 129-131:  “The study staff conducted the interviews in person at the participant households using appropriate COVID-19 infection prevention protocols, including masking and social distancing.” |
| 15. Whether efforts were taken to include those that were “Not met (n=32)”     a. No rapid nature but 32 | No, due to the rapid nature of survey and limited staff and budget to attempt multiple visits. |
| 16. The writing in this section has scope of improvement with respect to grammar and by avoiding repetitions of sentences. | We have made minor edits throughout to improve the clarify of the writing. |
| **Measurement and definitions**   17. Why was it decided to categorize vaccination status as partially if other brands were administered? Kindly provide justification for the classification used. were they classified? | The vaccination status was classified according to CDC guidance then as used in: <https://www.cdc.gov/mmwr/volumes/71/wr/mm7104e2.htm>  which we have now cited in the methods (lines 142) |
| 18. If the participants didn’t know the brand, how were they classified? | We added the following in methods in line 142-146: “If the vaccine brand was unknown and the first dose was missing, we classified the partial dose as missing, as determining partial vaccination status requires knowing the brand of at least the first dose. If the brand of either the first or second dose was missing, the full-dose status was considered missing, to avoid incorrect categorization. Finally, if all three doses were missing, the boosted status was also considered missing. ”  **Partial Dose Classification**   - **Logic:** Assigns a partial dose if only dose 1 is present (and it’s not "Johnson & Johnson") and both dose 2 and dose 3 are missing. - **Rationale:** This reflects a situation where only an initial dose has been received (excluding single-dose vaccines like Johnson & Johnson), so it makes sense to classify this as "partial." - **Handling Missing Values:** If dose 1 is missing, you set partial-dose to NA, since we can’t determine partial status without at least dose 1.   **Full Dose Classification**   - **Logic:** Assigns a full-dose in cases where:   - A single-dose vaccine ("Johnson & Johnson") is given, or   - Both dose 1 and dose 2 are present for vaccines requiring multiple doses. - **Rationale:** This captures the two main paths to full vaccination—either through a one-dose vaccine or a completed two-dose schedule. - **Handling Missing Values:** By setting full-dose to NA when dose 1 or dose 2 is missing, we avoid incorrectly categorizing incomplete as full.   **Boosted Dose Classification**   - **Logic:** Assigns a boosted status if:   - Johnson & Johnson was given as dose 1 and a second dose is available, or   - For multi-dose vaccines, all three doses (1, 2, and 3) are present. - **Rationale:** This classification captures the scenarios where a booster is given following either a single-dose primary vaccine or a completed two-dose series. - **Handling Missing Values:** Assigning NA if all doses are missing ensures that we don't assume a boost status without any dose information. |
| 19. Some of the parameters used to calculate vaccination accessibility don’t belong to this category (For example: “being aware if the nearest health facility had vaccine stock” comes under knowledge more than accessibility). Was this scale derived from a standard tool? If yes, provide details for the same. | We included this question to assess information accessibility—that is, how well information about vaccine availability was communicated to the community. In this context, local Female Community Health Volunteers (FCHVs) provided this information just before vaccination drives. This question was not derived from a standard tool but was introduced specifically to reflect the local context in Sarlahi, where FCHVs make announcements about vaccination availability. |
| **Data management and analysis**   20. Kindly mention how descriptive analysis was done – mean (SD) or frequency with proportions | I have added N(%) to each table and we mention “*^1^* n (%); Median (IQR)” in a footnote to Table 1. Added an edit to line 199 (Table 1). |
| 21. For inferential statistics – 95% CI and p value (how much is significant) | p<0.05 added on Table 4 footnotes. |
| 22. Regression – Better to report prevalence odds rather than odds ratio as this is a cross sectional study | In this study, we are reporting odds ratios rather than odds because we want to report the associations between exposures and outcome, not the odds of an event. In Table 4, we do provide the prevalences of the events along with the odds ratios. |
| **Results**   23. Table 1: The variables “Water source/latrine type”, etc are already accounted for in wealth quartiles. Mentioning these variables separately might not be appropriate while seeing for difference between completed vs incomplete primary series. If the author wants to retain these variables, they can create a separate table for basic socio-demographic characteristics and mention it there without comparing completed vs incomplete primary series. They can then include a few selected variables for the comparison aspect. | ‘Water source/latrine type,' along with other socio-demographic characteristics, are presented in Table 1 to enhance transparency for readers about the components of the wealth quintile composite. By including these variables, we aim to clarify the basis on which the wealth quintile categorization was constructed. However, these variables are not in the regression analyses as the reviewer is correct, that we would not want to include them as individual covariates, given they are already included in the wealth quintile variable.  We have also added overall Ns to table 1. |
| 24. Justification for including table 2 (Is there a need to highlight a separate table for vaccine card availability?) | The data did not fit in with other tables, but we think it is important to show card retention with respect to doses. |
| 25. Table 4: – Better to report prevalence odds; | Addressed above in #22 under Methods: Data management and analysis. |
| 26. How was social media usage measured? Has this variable been mentioned in earlier tables? | We have added this to table 1. |
| **Discussion**  There is scope of improvement in the structuring and flow of content in discussion. | We have made minor edits to the discussion to improve the flow. |
| 27. Details related to vaccine accessibility could be discussed with respect to other studies | Vaccine accessibility has been described in the primary outcome paper : <https://www.sciencedirect.com/science/article/pii/S0264410X24006613?via%3Dihub>  So, we added the following from line 284-287:  “Additionally, despite vaccine accessibility being reported as high in our study population, the majority did not know if vaccines were available in their nearest vaccination facility, indicating poor communication (17).” |
| 28. Content related to country of Bhutan can be cut short and relevant points to your study could be retained | We have shorten this in line 296-300:  “Bhutan, due to the temporary export halt of the AstraZeneca COVID-19 vaccine from India, decided to use mRNA vaccines, Moderna and Pfizer (35), for their second dose to complete the primary series. They were able to vaccinate more than 95% of their eligible population in the first two rounds of a vaccination campaign in 2021 (37).” |
| 29. Other biases with respect to sampling and measurement could be elaborated and its implication discussed. | We mention in our limitation the lack of generalizability due the sampling method:  Line 304-312:  “The study was conducted in four municipalities, which roughly approximate the larger Terai region, but is not necessarily representative of Nepal overall. This study utilized a pre-existing census from the parent randomized trial as the sampling frame that comprised households that had a woman 15-30 years of age. Hence, our study households may not reflect all households in the district, although most households, which include multigenerational families, have women of childbearing age. Our study population had a higher proportion of females, as males tended to be working (farming or other) during the day or working overseas. Hence, this gender imbalance may contribute to a lack of generalizability.” |
